# Health Impacts of the Stay-at-Home Order on Community-Dwelling Older Adults and How Technologies May Help: Focus Group Study

**DOI:** 10.2196/25779

**Published:** 2021-03-22

**Authors:** Jessica R Daly, Colin Depp, Sarah A Graham, Dilip V Jeste, Ho-Cheol Kim, Ellen E Lee, Camille Nebeker

**Affiliations:** 1 Department of Psychiatry Sam and Rose Stein Institute for Research on Aging University of California, San Diego La Jolla, CA United States; 2 AI and Cognitive Software, IBM Research-Almaden San Jose, CA United States; 3 Research Center for Optimal Digital Ethics in Health Herbert Wertheim School of Public Health and Human Longevity Science University of California, San Diego La Jolla, CA United States

**Keywords:** aging, quarantine, mental health, physical health, social isolation, COVID-19 pandemic, continued care senior housing community, CCSHC, qualitative research, videoconferencing, older adults, gerontechnology, loneliness, housing for the elderly, independent living

## Abstract

**Background:**

As of March 2021, in the USA, the COVID-19 pandemic has resulted in over 500,000 deaths, with a majority being people over 65 years of age. Since the start of the pandemic in March 2020, preventive measures, including lockdowns, social isolation, quarantine, and social distancing, have been implemented to reduce viral spread. These measures, while effective for risk prevention, may contribute to increased social isolation and loneliness among older adults and negatively impact their mental and physical health.

**Objective:**

This study aimed to assess the impact of the COVID-19 pandemic and the resulting “Stay-at-Home” order on the mental and physical health of older adults and to explore ways to safely increase social connectedness among them.

**Methods:**

This qualitative study involved older adults living in a Continued Care Senior Housing Community (CCSHC) in southern California, USA. Four 90-minute focus groups were convened using the Zoom Video Communications platform during May 2020, involving 21 CCSHC residents. Participants were asked to describe how they were managing during the “stay-at-home” mandate that was implemented in March 2020, including its impact on their physical and mental health. Transcripts of each focus group were analyzed using qualitative methods.

**Results:**

Four themes emerged from the qualitative data: (1) impact of the quarantine on health and well-being, (2) communication innovation and technology use, (3) effective ways of coping with the quarantine, and (4) improving access to technology and training. Participants reported a threat to their mental and physical health directly tied to the quarantine and exacerbated by social isolation and decreased physical activity. Technology was identified as a lifeline for many who are socially isolated from their friends and family.

**Conclusions:**

Our study findings suggest that technology access, connectivity, and literacy are potential game-changers to supporting the mental and physical health of older adults and must be prioritized for future research.

## Introduction

Social isolation and loneliness affect human health, well-being, and overall quality of life [1] and are known risk factors for poor mental and physical health across an individual’s lifespan, particularly among adults aged over 65 years [2-4]. Social isolation is defined as “the objective lack or limited extent of social contacts with others,” whereas loneliness is defined as “the perception of social isolation or the subjective feeling of being lonely” [5]. Research has documented the relationship between social isolation and loneliness and mental health contributing to suicides and opioid-related deaths [6]; poor physical health, including cardiovascular disease and stroke [7]; and premature mortality [8].

The serious societal and public health concerns around social isolation and loneliness prompted the American Association for Retired Persons to commission the National Academies to convene a working group to address social isolation and loneliness among older adults. The resulting report, which included recommendations for improving our health care system, was published in February 2020, just around the time when the COVID-19 pandemic was beginning to grip the United States. The COVID-19 pandemic and related social distancing guidelines are expected to exacerbate social isolation and loneliness, particularly among older adults [9]. In fact, the “double pandemic” of social isolation and COVID-19 has received increasing attention, as the balance of survival against the sacrifices of social connectedness are clearly at odds [8]. An important consideration is how long preventive measures can be kept in place, while considering the consequences of such measures on the public’s health and well-being.

In March 2020, “Stay-at-Home” quarantine orders were imposed across the USA to mitigate the spread of COVID-19. As Continued Care Senior Housing Community (CCSHC) residents were considered at high risk of infection, conservative protocols were implemented to prevent the escalation of infection and deaths among older adult residents. The Centers for Disease Control and Prevention issued guidance for CCSHCs to increase protections for residents and workers and advised administrators to work with their local health officials to develop safe and feasible guidelines [10]. Recommendations included risk reduction practices to limit exposure by closing access to common areas (eg, gyms and restaurants) and encouraged workers and residents to refrain from visiting other individuals' living spaces. In addition, those residing or permitted to work within the facility were asked to maintain at least six-feet physical distance, complete daily health screens, and wear face coverings. In many cases, nonessential workers and visitors were not permitted within the facilities.

As COVID-19 continued to spread, it was clear that older adults were at significant risk, particularly those living in CCSHCs where outbreaks were concentrated and associated with community spread and a higher death rate. In the United States, there are nearly 2000 CCSHCs offering long-term care options for older adults [11]. We selected a CCSHC in southern California for our qualitative study designed to assess the impact of quarantine on resident’s physical and mental health.

This CCSHC was selected because many of its residents are currently involved as participants in an ongoing longitudinal research study. This larger parent study is designed to learn about cognitive, physical, and mental health and factors that influence healthy aging among older adults living independently in a CCSHC [12]. The community-based longitudinal study involves 112 participants (about one-third of all the CCSHC residents) aged 65-100 years and living independently. For our qualitative study, we invited residents, including participants of the longitudinal study, to participate in one of four virtual 90-minute focus group meetings scheduled for May 2020. The aim of the study was to assess the impact of the COVID-19 pandemic and resulting quarantine on the residents’ mental and physical health and to explore ways to safely increase social connectedness among them.

## Methods

### Inclusion Criteria

Four focus groups were conducted in May 2020 using the Zoom videoconferencing platform; the focus groups comprised residents from a CCSHC in southern California aged 65 years and above. Residents were considered eligible for this study if they were an independent-living resident at the CCSHC; willing to participate in a 90-minute group discussion using the Zoom videoconferencing platform; had access to the Zoom platform using a phone, tablet, or computer; and had a general understanding of how to use the platform.

### Recruitment and Scheduling

The study was reviewed and verified as exempt by the institutional review board. An informational flyer was circulated electronically and physically to residents. The research staff also contacted participants of the ongoing longitudinal study to share study information and gauge their interest. Interested residents were provided with details of the focus group (eg, date and time) and asked to indicate their availability. A research assistant confirmed the selected date/time along with instructions for joining the meeting via the Zoom platform. Two email reminders were sent prior to the focus group sessions with the meeting link and a message that technical support was available for 30 minutes prior to the meeting.

### Data Collection: Focus Group Semi-Structured Interview Guide

Participants joined the Zoom focus group via a phone, tablet, or computer device, and any technical difficulties they experienced were addressed by the research staff. Each 90-minute session was led by a trained focus group facilitator (CN or JD), with both facilitators attending all four sessions.

Focus groups began with an introduction to the study purpose, confidentiality expectations, and ground rules. All focus group attendees gave verbal consent to participate and the meeting to be recorded. The first focus group took place on May 5, 2020, that is, 47 days after the stay-at-home order was issued in California. The fourth or the last focus group was held on May 27, 2020, that is, 22 days after the first group and 69 days after the stay-at-home mandate was imposed.

The focus group protocol items (see Table 1) included two forced choice and several open-ended prompts to gauge participants’ responses to the quarantine measures. The polling feature of the Zoom platform was used early in the session to have participants anonymously respond to two questions that asked how the social distancing requirement had impacted their mental health and physical health. The facilitator also asked open-ended questions to explore how the quarantine was affecting participants’ mental and physical health, thoughts about what other residents might be experiencing, strategies to manage stress and isolation, the use of technology to increase social connectedness, and potential solutions that would mitigate health consequences due to quarantine.

**Table 1:** Focus group protocol items.

Category	Question
Zoom polling feature	The impact of the corona virus pandemic social distancing requirement has impacted my mental health: Not at all Somewhat A lot The impact of the corona virus pandemic social distancing requirement has impacted my physical health: Not at all Somewhat A lot
Social distancing	How would you describe the impact of social distancing on your (a) mental health and (b) physical health? What about the people around you?
Strategies	What strategies have you found helpful to manage your anxiety and reduce stress? What strategies have your family or friends used?
Activities	Have you engaged in new activities to help cope with the coronavirus pandemic?
Technology	How are you using technology differently than pre-COVID?
Intervention	We are planning to conduct a program that, we hope, will reduce loneliness in senior housing communities. What are your thoughts about this type of program, and would you be interested?

### Community Validation

Two additional group meetings were held in September 2020 with a subset of participants who reviewed a summary presentation of the study results. One of the objectives of this meeting was to check in with the participants, also called “member checking,” to validate that what they learned from the focus group meetings was consistent with their recollection and to confirm the trustworthiness of findings [13]. The participants also provided an update on how their circumstances had changed in the 3 months since participating in the focus groups.

### Data Analysis

Group discussions were digitally recorded and professionally transcribed. Transcripts were deidentified by redacting identifiable information. Two researchers (CN and JD) manually coded and analyzed the transcripts to identify distinct themes and patterns. The analysis followed a traditional content analysis process whereby text was reviewed, codes were developed, and themes and patterns were identified [14]. The inductive coding framework was developed from the interview guide and research questions. Both researchers (CN and JD) reviewed notes taken during each focus group, along with all transcripts, and independently coded two transcripts that were then compared and analyzed to verify intercoder consistency. Codes were then grouped into categories, and emerging themes were identified. The resulting codebook was used to guide the analysis of the remaining focus groups’ transcripts carried out by JD.

## Results

### CCSHC Campus Management Response to COVID-19

When the California stay-at-home order went into effect on March 19, 2020, the CCSHC management effected the following changes: (1) closed the restaurant and initiated delivery of meals directly to each resident’s apartment; (2) closed the gym and suspended organized social gatherings (eg, bingo, trivia, and exercise classes); and (3) closed the campus to all external visitors except for deliveries.

#### Focus Groups

Twenty-one residents participated in one of four 90-minute focus group meetings. Demographic information was available for 20 of the 21 participants who were also enrolled in the parent study. Participants were predominately Caucasian (20/21, 95%) and female (14/21, 67%) with a mean age of 80 (SD 5.5) years. A majority of the participants (17/21, 81%) had attained an associate degree or higher, with 86% (18/21) reporting an annual household income over US $50,000.

#### Poll Results

Impact of social distancing on participants’ mental and physical health was quantified from poll responses (see Table 1). Of the 21 participants, 17 (81%) confirmed they experienced a negative impact on their mental health and 12 (57%) indicated they experienced a negative impact on their physical health.

### Results of the Thematic Analysis

Four themes were identified from the qualitative analysis and labeled as follows: (1) impact of the quarantine on health and well-being, (2) communication innovation and technology use, (3) effective ways of coping with the quarantine, and (4) improving access to technology and training. Qualitative responses attest to the effects of quarantine on the participants’ mental and physical health, which were attributed to restricted social routines, including limited access to family and friends, and reduced physical activity.

#### Impact of the Quarantine on Health and Well-Being

A common frustration expressed across all focus groups was the negative impact of the quarantine on participants’ physical and mental health. Negative effects most frequently mentioned included weight gain, decreased mobility, worsening of existing health conditions, and/or development of new conditions due to decreased physical activity.

I miss the gym a lot. I was there almost every day, and my mobility has suffered from not being able to use the machines down there. I'm limited in the distance that I can walk due to neuropathy and things like that, arthritis, so it's really taken a toll.

The not being able to use the equipment down in the gym has really been detrimental to my physical health because I have these stents in my legs, and the one thing that my doctors told me I needed to do was to be on a treadmill or on an exercise bike several times a week for an hour a day and I haven’t been able to...

Participants expressed a consistently strong preference for the gym to reopen and provided ideas for how the management could reopen the facility safely. Some suggested that gym visits could be scheduled and a sanitation protocol could be included, which they felt was feasible since the facility was not open to the public. To mitigate the reduced access to the gym, the management organized 15-minute group workout classes that were conducted by a health educator in the parking lot. Participants could join from their balconies or using a web-based platform. Although many appreciated the opportunity to participate, it did not replace their desire to return to the in-house gym facility.

From a mental health perspective, limited opportunity to engage with others was taking a toll. Participants expressed their desire to interact with friends and family and hoped for a return to the routines and activities that originally connected them as a community. Many emphasized a longing for physical touch (eg, hugging) and social connection without a mask, so they could interact with smiles and other facial expressions. Additionally, participants expressed how much they missed celebrations, and some felt diminished joy for life. Participants who had lost their partner prior to the quarantine spoke about increased feelings of grief associated with being alone. Several participants described short bouts of anxiety and depression. Some managed these low moods by going for a drive, working on a hobby, or enjoying the company of their partner or pet. Others worried that the impact of the quarantine on their mental health may be long lasting.

I don't normally get real [sic] depressed. But, for about 2 days, I just really thought this is it, that we're never gonna get out of here. Because I wanna keep things closed down 'til it's really safe. I don't feel good about going out and doing things...

It's all of these quarantine fears that have cast question over my capability to handle myself, which has been an ever-present piece of fear in my mind...It's all causing a fearful mentality of what could happen.

For many participants, social connectivity and group activities were key reasons for relocating to the CCSHC; however, the lockdown requirements were met with frustration, despite understanding the need to stay safe during the pandemic.

I lost human connection and I'm the type of person that wants to be with a group of people and do fun things together. We lost our family connection, family celebration, family hugging, joking around, and sometimes I feel lost.

In addition to physical distancing requirements, the use of face masks contributed to the loss of connection. One participant noted that some older adults rely on reading lips to follow a conversation, particularly when experiencing hearing loss. With masks, they have fewer social and emotional signals, making conversing in person more difficult. The inability to see a smile or other forms of expression proved challenging when physically near one another, as depicted by this participant.

I'm kind of a touchy-feely person and enjoy looking at people's faces and expressions and the tone of their voice, so it's been a big loss.

#### Communication Innovation and Technology Use

Communication innovation reflects creative strategies for social engagement, including technology use, that participants developed to mitigate the social isolation stemming from the quarantine. Most participants used a smartphone, and some used a videoconferencing platform.

We use a lot of texting and phone calls to get back and forth and sending pictures back and forth, so it helps a lot. It makes a big difference.

Video communication platforms (eg, Zoom and Skype) were identified as an ideal solution for connecting with family and friends while in quarantine. Several participants mentioned using these tools for virtual book clubs and social groups. They also enjoyed the idea of leveraging technology to support access to discussion groups with specific topics, interactive group games, and/or support groups.

I think that there needs to be some appreciation and recognition that Zoom is a tool that can be very beneficial for this community on an ongoing basis.

There's so much to be said in a face and an expression and the quality of voice [sic]. It just communicates so much, while safely using videoconferencing

to connect

One participant coordinated web access to her church for Sunday services, and another continued participating in her women’s group via Zoom. The use of digital medicine was also mentioned as an advantage of the quarantine, making it possible to speak with a clinician without having to travel to the clinic themselves.

My women's group that I belonged to for 25 years is scheduled for this afternoon and is supposed to be on Zoom. We have sort of grown up together and it's a support group and a friendship group…

Many participants were concerned about the shy or reclusive residents who may not have well-established relationships within the community and felt that community videoconference meetings could help to connect these individuals with the rest of the community and reduce the effects of social isolation and loneliness.

I was thinking that Zoom is a way for introverts to reach out from the safety of their own home and sometimes having an anchorage available gives courage to movement. And that being safe in your own apartment and reaching out is a little step that is safer to take than putting your whole self out there.

Although technology was seen as an ideal intermediary resource, participants preferred interacting with other residents while physically distancing, for example, meeting for a meal in the parlor or walking around the campus grounds.

There are 6 to 8 of us that sit in one of the parlors and have dinner together, although we're all at our own tables and we’re all at least 6 feet apart...

#### Effective Ways of Coping with the Quarantine

The two most common ways that the participants coped with the quarantine was by maintaining a positive attitude and finding support and companionship within the community. As previously mentioned, technology was also used as a coping mechanism to provide social connection and access to certain activities (eg, church).

With respect to attitude, many participants recognized that they had some control in how they perceived the quarantine as either an opportunity or a threat. One participant stressed that her framing of the situation made a big difference in her perspective of the pandemic and reduced its negative impact.

It's all about attitude, I think. I saw something recently that said we can look at ourselves as being stuck at home or we can look at ourselves as being safe at home. So, to me, a lot of it is just in how you look at it. We are safe, we're healthy, and this will end.

Similarly, another participant noted that in addition to recognizing how you can control your own narrative, you can also let others know that they have the ability to change their attitude and, subsequently, their reality.

Finding safe community support was another tool that participants mentioned. Within the CCSHC, each building or floor is considered an individual neighborhood. Participants described different ways that their “neighborhoods” supported one another. For instance, singing or reciting the pledge of allegiance every day at noon was becoming a custom.

Most of our residents come out every day at noon, and we say the Pledge of Allegiance and sing a song. And then we spend just a few minutes making sure everybody is okay and talking about any problems they might have come up with...everybody says it's really good for their mental health to see each other.

Another neighborhood held a fundraiser by selling ice cream bars down the hallways, while distancing and wearing proper protective equipment. Other neighborhoods posted artwork near the elevators for their fellow residents to enjoy. Many found happiness in celebrating uncommon holidays, such as National Pretzel Day on April 26, 2020. Residents left bags of pretzels at their neighbor’s doorsteps with small notes attached, hoping that this would bring some celebration back into their lives.

On the positive side, it seems to me that floors have become little neighborhoods and are beginning to look out for each other more than they used to.

Participants who had a pet expressed gratitude for the companionship and the additional motivation to walk outside, which supported their physical activity.

But if you have a dog or a cat...there is company in that too. That helps a lot. But I think it would be very hard if you're by yourself in your apartment.

#### Improving Access to Technology and Training

Despite mentioning technology as a communication coping strategy to mitigate the effects of social isolation and loneliness, participants emphasized that technological barriers existed. For those with access, the cost of an internet service was potentially prohibitive. For those with both a device and connection, training was needed to learn how to use technology.

In our meeting earlier today, they [management] said that only about 50% of the residents here are computer literate.

Participants suggested that a peer-educator model, whereby participants taught each other, would be desirable. This approach could facilitate a safe and enjoyable environment that would enable them to interact and learn how to use their devices.

I like the idea of more Zoom activities for meetings and things like that. The one thing that is needed, though, is a concerted effort to educate people and help them set their computers up.

### Debriefing Meetings

Two additional meetings held in September 2020 included a subset of participants to validate the findings. Participants confirmed our study results and, similar to the findings in May 2020, they remained frustrated, and negative impacts of the quarantine on their mental and physical health persisted. Many appreciated that restrictions were loosening and felt encouraged by the reopening of the community gym and local restaurant, yet the monotony of day-to-day reality was taking a toll on their mental health. Social activities had not resumed and community facilities where people gathered (eg, dining rooms and clubs) remained closed. Participants expressed concern that the gym may be closed again if the county community outbreak numbers increased, which would be detrimental to their physical health and mental well-being.

They [management] failed to weigh the effects that the re-closing of the gym would have on the residents, you know. It's getting to the point where not having a gym is just as bad as the risk of contracting the virus, so there has to be a trade.

Participants continued to use technology (eg, Zoom, videoconferencing, and texting) as a communication coping strategy but reiterated their need for physical contact, emphasizing the negative effects of diminished social contact with friends and family. Participants generally agreed that the primary source of frustration came from the quarantine having “no end in sight.”

Additionally, they felt the standards and guidelines within their community lacked detail, and it was unclear what activities were allowed. Despite these frustrations, participants continued to focus on potential solutions, and again expressed the need for simple and straightforward technology training, emphasizing the peer-educator model, and the potential for videoconferencing to aid social connection.

## Discussion

### Principal Findings

This focus group study of CCSHC residents informs our understanding of the impact that the COVID-19 pandemic and the resulting quarantine and increased social isolation has had on older adults’ physical and mental health. Additionally, the role of technology emerged as being key to preserving social connectedness for many within these communities; however, unfortunately, the technology was not accessible by all participants. Below, we discuss the major findings of the focus groups with recommendations for next steps.

### Impact on Physical and Mental Health

Prior to COVID-19, older adults already represented the least physically active age group in the United States [15]. Greater self-reported physical activity has been associated with better self-reported and objectively measured physical health of older adults living in a retirement community [16]. Reduced physical activity may also have negative implications for other aspects of health such as cognitive trajectories; older adults that spend less time in physical activities of moderate-to-vigorous intensity may have a greater long-term risk for cognitive impairment [17]. It was mentioned multiple times by participants that physical and mental health were directly tied to one another and when one declined, the other did as well. These assertions are supported by recent literature demonstrating that even light physical activity during the COVID-19 pandemic may help alleviate the negative impact on the mental health of older adults who are socially isolated [18]. Furthermore, studies have reported that decreased physical activity may increase susceptibility of at-risk groups to infections and exacerbate existing chronic medical conditions such as cardiovascular disease and cancer [19]. Alternative exercise and social activities are clearly necessary to maintain the physical and mental health and well-being of socially isolated older adults.

Prior studies have associated social isolation and loneliness itself with poor physical and mental health outcomes. Specifically, social isolation and loneliness have been associated with unhealthy lifestyle behaviors, including increased smoking, alcohol consumption, and malnutrition [20-23]. Moreover, social isolation and loneliness are independent predictors of depression, anxiety, and cognitive impairments among older adults [24-26]. The duration of social isolation and loneliness is also of concern, particularly in conjunction with quarantine, as recent findings suggest that social disengagement and fewer subjectively meaningful interpersonal interactions are related to decreased physical performance over time [27]. Evidently, more research is needed to assess the impact of quarantine-induced social isolation and loneliness, with an emphasis on how technology may be useful in reducing health risks.

### Technology Solutions

Participants highlighted technology as critical to maintaining social connectedness, by using Zoom and other media to reduce social isolation and loneliness throughout quarantine. The barriers to adoption of technology reported by the participants were consistent with those reported in prior research among older adults, including attitudes, cognitive ability, prior experience, product design, cost, and access [28,29]. An issue with the design of health technologies (eg, monitors and sensors, communication systems, and artificial intelligence), is that older adults are rarely involved in the design process or are engaged too late in the development cycle. For example, older adults are the likely major users of telehealth, yet little attention is given to their end-user perspectives [30]. Older adults may therefore have a lack of confidence that they can learn to use these technologies, or they may have limited interest [28,31]. Prior research has shown that older adults want to assist with co-designing health technologies and can help technology developers understand how to design, considering their physical and cognitive limitations as well as privacy concerns [32].

### Importance of Attitude

Aging is associated with heterogeneity, with some older adults having increased emotional responses to naturally occurring daily stressors and others being more resilient [33]. Increased resilience has been associated with better cognitive function and self-perceived health, reduced depression, and greater optimism for older adults [34]. Negative stereotypes associated with aging, particularly negative self-perception of aging, may play an additional role in the varying severity levels of the effects of quarantine on the mental health of older adults [35]. Research has shown that negative attitudes towards aging can increase emotional response to daily stressors [36]. Our study found that maintaining a positive attitude during quarantine was a key coping factor. The combined effects of varying resiliency and attitudes towards aging among older adults are likely responsible for the diverse findings in recently published studies evaluating the impact of the COVID-19 pandemic on senior persons [37].

Participants demonstrated compassion for other residents in many ways (eg, regular check-ins). This compassion for others may relate to the community prioritizing supportive actions, where neighbors were “beginning to look out for each other more than they used to.” These findings support the role of compassion in battling potential loneliness caused by social isolation [3,38,39] and reinforce the need for interventions to reduce loneliness by promoting compassion [40,41]. Once technology barriers have been addressed, web-based platforms could be used as a resource for these types of interventions.

A final and concerning observation was that the fear of exposure to COVID-19 infection among the participants appeared to diminish over time, while feelings of quarantine frustrations increased. Being “safe at home” comes with frustrations that could undermine the ability to persist with behaviors that support infection prevention; subsequently, the behaviors enacted due to these frustrations may have contributed to the second COVID-19 wave in the USA that occurred in fall 2020.

### Recommendations for Addressing Social Isolation During COVID-19

The COVID-19 pandemic will continue to exacerbate social isolation and loneliness and related health issues for older adults. There are actions that individuals and organizations, including CCSHCs, can undertake to mitigate quarantine harms and related social and physical implications. Due to the current quarantine restrictions, technology emerged as a clear and viable solution for supporting older adult’s mental and physical health while practicing social distancing. The key areas that must be prioritized for future research are described below.

#### Communication Platforms

Recognizing the prevalence of and predictors for loneliness in older adult populations is important. A grim statistic in a survey of older adults (N=6,786) revealed that 39% suffered from loneliness, with 5% of the total population reporting loneliness often or always [42]. Given that loneliness is linked to social isolation, could loneliness be prevented through proactive interventions? One suggestion from the study participants was to schedule more frequent and consistent virtual gatherings using communication platforms (eg, Zoom and Google Hangouts). These gatherings could bring the community together for physical activity, educational lectures, book clubs, and other social opportunities. Participants suggested that access to these virtual opportunities may encourage access by people who normally do not attend social activities, as they would be able to attend without the pressure to participate. Online group gatherings may hold promise for older adults, as current research shows that by implementing a group intervention reduced social isolation and loneliness and health care costs, whereas it increased feelings of well-being [43]. Access to resources that facilitate physical activity were prioritized by participants to support their physical and mental health, which is known to alleviate the negative consequences of quarantine [18]. Increased efforts to provide older adults with consistent and easily accessible workouts, either via web-based platforms or by observing social distancing within the community, are essential to support their overall well-being.

#### Access and Connectivity

Access to technologies, including communication platforms and social robots, may mitigate frustrations associated with social distancing and isolation. Presently, little is known about the extent to which campus residents have access to or own personal technologies. Assessing interest among the participants as well as facilitators and barriers to access are the first steps for research in this direction. Results could inform interventions to connect with and use technologies. If technology is a key to the short- and long-term health and well-being of older adults, efforts should be made by programs of all levels to identify needs and bridge gaps so that participants are able to access and use technologies that support social connection with friends, family, and the community. Additionally, when information is communicated by the management using a technology platform that not all can access, the leadership is encouraged to explore all avenues necessary to reach residents, as communication is a lifeline for many during these uncertain times.

#### Technology Literacy

Once challenges pertaining to access and connectivity are addressed, which is an issue of digital equity and beyond the scope of this paper, training to use the technology is necessary. In this study, participants expressed a lack of confidence that may be addressed through education; however, with the pandemic raging, it is challenging to match appropriate education to promote technology literacy. Participants suggested that the development of a peer-led education program would be a desirable method, as it would increase community capacity safely. The peer-educator model suggested by participants has been used successfully in programs designed for older adults [44,45]. Although this is a starting point, these resources will need to be evaluated, augmented, and implemented to support older adults, particularly those with limited technology experience.

### Limitations

There are several limitations to this study. Focus groups were conducted using the Zoom videoconferencing platform, rather than in person, due to the quarantine restrictions. Participants represented a select subset of CCSHC residents who had technology access, connectivity, and some knowledge of how to navigate the technology. The majority of participants were highly educated, financially stable, Caucasian, and represented a single CCSHC in southern California. For these reasons, the results of this study may not be generalizable to other locations and more socioeconomically diverse older adult populations.

### Conclusions

This study engaged residents of a CCSHC to learn how the COVID-19 pandemic and the resulting quarantine has impacted the mental and physical health of older adults. The preventive measures to reduce the spread of infection has revealed that technology access, connectivity, and literacy are potential game-changers in supporting the mental and physical health of older adults and must be prioritized for future research. CCSHC residents reported the impact of the quarantine on their mental and physical health was directly tied to the stay-at-home mandate due to social isolation and reduced physical activity. Technology was identified as a lifeline for many participants who were socially isolated from their friends and family. The digital divide is more prevalent among older adults than other populations and will exacerbate health disparities if not prioritized and addressed.

## Abbreviations

CCSHC: Continued Care Senior Housing Community
